# Camera Calibration for Water-Biota Research: The Projected Area of Vegetation

**DOI:** 10.3390/s151229798

**Published:** 2015-12-03

**Authors:** Rene Wackrow, Edgar Ferreira, Jim Chandler, Koji Shiono

**Affiliations:** School of Civil and Building Engineering, Loughborough University, Loughborough, Leicestershire LE11 3TU, UK; e.a.ferreira@lboro.ac.uk (E.F.); j.h.Chandler@lboro.ac.uk (J.C.); k.shiono@lboro.ac.uk (K.S.)

**Keywords:** photogrammetry, underwater imaging, eco-hydraulics, flow visualization and imaging, laboratory studies, plants reconfiguration, vegetated flows

## Abstract

Imaging systems have an indisputable role in revealing vegetation posture under diverse flow conditions, image sequences being generated with off the shelf digital cameras. Such sensors are cheap but introduce a range of distortion effects, a trait only marginally tackled in hydraulic studies focusing on water-vegetation dependencies. This paper aims to bridge this gap by presenting a simple calibration method to remove both camera lens distortion and refractive effects of water. The effectiveness of the method is illustrated using the variable projected area, computed for both simple and complex shaped objects. Results demonstrate the significance of correcting images using a combined lens distortion and refraction model, prior to determining projected areas and further data analysis. Use of this technique is expected to increase data reliability for future work on vegetated channels.

## 1. Introduction

The capability of aquatic plants to deform and “reconfigure” is critical to the functioning of lotic ecosystems [1,2]. Specifically, adverse effects imposed by these barriers (in terms of flow resistance) are counterbalanced by a variety of ecosystem services associated with plant motion, namely regulating services [3]. Thus, some authors have sought to quantify plants’ morphology as a way to assess the performance of different species [4].

Sagnes [4] describes the technical challenges with quantifying the frontal area of a plant. Specifically, Sagnes identifies that “the projected frontal surface area (*A_f_*) captures flow-induced shape variation and is seemingly the most realistic physical description”. Different setups and image perspectives have been adopted to estimate *A_f_* or equivalent descriptors, ranging from: mirrors attached to the bottom part of laboratory facilities or *in situ* environments combined with top view images using regular cameras [4,5,6]; images acquired in still air [7] and water conditions [8,9]; to submerged digital cameras aligned with the plant mass centre [10,11]. If light absorption or scattering is not dominant in the course of image acquisition, underwater techniques provide the only opportunity to accurately inspect the morphological reconfiguration of vegetation specimens in the field. Nevertheless, non-metric sensors such as consumer grade digital cameras do not possess, as opposed to photogrammetric or metric cameras, a calibration certificate. Basically, this demands deriving a set of parameters which can be used to describe the internal geometry of the imaging system (e.g., focal length, principal point offset, and radial and tangential lens distortion) [12]. This step is crucial, notably if precise spatial information is to be extracted and carried out through a process known as “self-calibrating bundle adjustment” [13,14]. The impact of lens distortion on subsequent measurements have been previously mentioned on vegetated studies, but have been neither thoroughly investigated nor quantified. For instance, Jalonen *et al.* [11] identified that scaling errors can distort the estimated projected area up to 10%, however, it is a plausible conjecture that these results possibly include a combination of errors caused by scale constraints and uncorrected lens distortion. Even in the work conducted by Sagnes [4], possibly the most comprehensive work on the topic that one can find in the literature, overlook this aspect. Our belief is that this is mainly a consequence of user unawareness of imaging geometry or a procedure to appropriately calibrate non-metric imagery. Whittaker [15] states that in the absence of a known focal length, distortion effects cannot be scrutinized and Wunder *et al.* [10] assumed, without apparent reason, that camera distortion effects were minimized in their work. Bearing in mind these considerations, this paper presents a method based on well-established photogrammetric principles to eliminate lens distortion in both dry and wet environments and compares projected areas using non-calibrated and calibrated cameras. Our work proves that a simple methodology, easily adoptable by experimentalists, allows for an effective camera calibration, thus enabling refinement of existing experimental protocols, particularly those prevailing in laboratory-based activities. The present analysis is restricted to the parameter projected area due to its relevance in aquatic studies (e.g., to evaluate the drag coefficient) but conclusions stemming from this work are equally valid for other morphological studies using similar imaging systems.

Tests performed for this work are explained in the next section. Afterwards, the camera calibration procedure is described. Finally, results are presented and some conclusions drawn.

## 2. Experimental Setup

**Figure 1:**
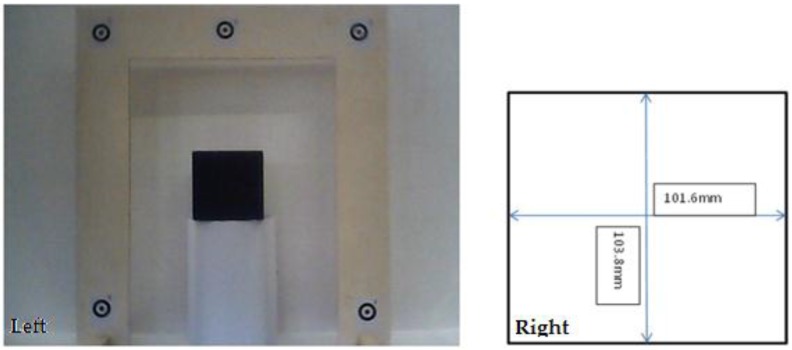
Metal cube and target frame used to provide a reference area (**Left**) and respective dimensions of the cube (**Right**).

**Figure 2:**
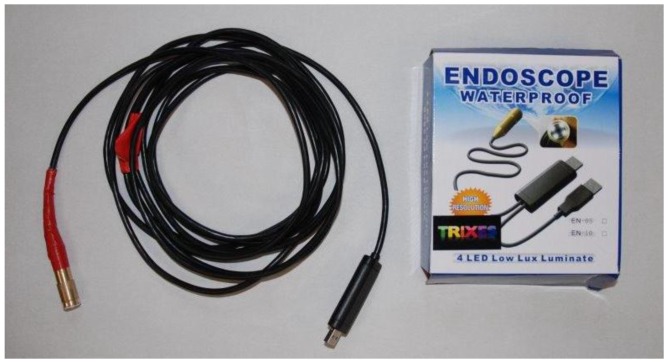
Underwater endoscope camera (resolution: 640 × 480 pixels; price July 2013: £25).

Three different experimental setups were employed to determine the projected area of an object in dry conditions and in both submerged static and submerged flow conditions (discharge: 0.124 m^3^·s^−1^, water depth: 0.275 m, flume length: 5.24 m, flume width: 0.915 m). Areas evaluated in these practical applications included the use of a simple metal cube, which provided an accurate reference area (0.01055 m^2^), and a real plant (bush species: Buxus sempervirens, height: 0.20 m). In all these measurements, distances between photogrammetric targets attached to a wooden frame (Figure 1) were determined using a vernier calliper, and used for scaling purposes in the process of calculating *A_f_* using digital imagery and photogrammetric measurement. The target frame was located in the same plane as the front of the test object. A video sequence of the objects surface area *A_f_* was acquired using an underwater endoscope camera (Figure 2), at an object to camera distance of 0.7 m, and approximately perpendicular to the metal cube and vegetation bodies.

**Figure 3:**
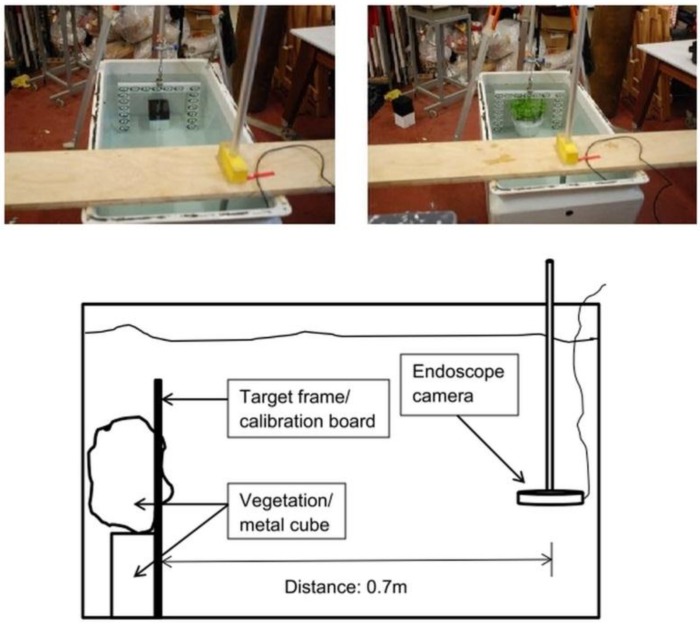
Plastic water tank setup.

**Figure 4:**
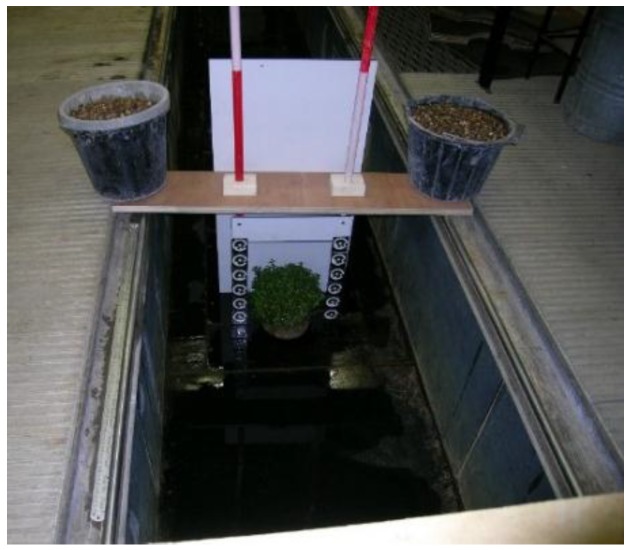
Open-channel flume.

The trial was conducted under dry conditions and furthered the opportunity to test the methodology without the additional distorting effect of the light rays passing through water due to refraction. Furthermore, for this attempt, a DSLR camera (Nikon D80, resolution: 3872 × 2592 pixels), shown to be suitable for accurate photogrammetric measurement in the past [16], was also employed for comparing images taken by both cameras. Use of a plastic water tank (submerged static conditions) offered a controlled environment to calibrate the underwater camera and assess if the lens distortion and refractive effects due to the water could be accurately modelled (Figure 3). Results achieved using the plastic water tank encouraged a further test to determine the projected area of both objects, *i.e.*, the cube and the bush, under flow conditions in an open-channel flume (Figure 4).

**Figure 5:**
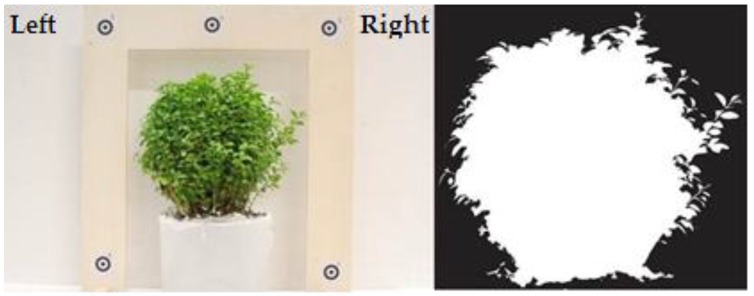
Bush and reference frame used for area determination (**Left**) and binary image obtained from Matlab (**Right**).

For the three cases mentioned above (still air, unstressed and stressed conditions), a Matlab routine was developed to manipulate and measure the images containing the object and the target frame (for stressed flow conditions an image was arbitrarily selected from the video footage). After reading the image file, a Matlab function was used to measure the distance between two photogrammetric targets in the image space. The measured distance in the image space and the distance measured in the object space were used to calculate an image scale factor. In essence, the routine converts an RGB image to a binary image using a simple 2-fold image classification. The pixels in the region of interest (*i.e.*, pixels representing the cube and the bush) are represented by white pixels, whilst all other objects are represented by black pixel values (Figure 5). Pixels representing the cube and the bush were counted automatically and the area was quantified by using the image scale factor. When attempted, image thresholding is almost certainly affected by some degree of uncertainty/imprecision (for example, *A_f_* is slightly overestimated in Figure 5). Hence, in practical terms, each researcher should carry out a systematic modification of the threshold value until the desired classification is reached.

It needs to be recognized that most cameras are not designed for accurate photogrammetric measurement [17]. Camera lenses are characterized by significant lens distortion which degrades the achievable accuracy in the object space [18] and additionally, in our study, the distortion effects of the endoscope camera will also change radically when used in an underwater environment as a result of water refraction. Such imaging sensors can be calibrated to minimize the combined effect of these two phenomena, *i.e.*, lens distortions and refraction effects for a specific camera to object distance. Routinely, this is done by assuming that these two components are implicitly considered in the distortion terms of the functional model known as the extended collinearity equations [19,20]. The camera calibration process that has been used prior to computing *A_f_* in both the dry and underwater studies constitutes the core of this work and is portrayed in the subsequent section.

## 3. Camera Calibration

The extended collinearity equations provide a framework to directly transform the object coordinates into the corresponding photo coordinates [21,22]

(1) $$\begin{array}{l} {\text{x′}}_{\text{a}}={\;x}_{\text{p}}-\text{c}\cdot \frac{\left[{\text{r}}_{11}\left({\text{X}}_{0}-{\text{X}}_{\text{A}}\right)+{\text{r}}_{12}\left({\text{Y}}_{0}-{\text{Y}}_{\text{A}}\right)+{\text{r}}_{13}\left({\text{Z}}_{0}-{\text{Z}}_{\text{A}}\right)\right]}{\left[{\text{r}}_{31}\left({\text{X}}_{0}-{\text{X}}_{\text{A}}\right)+{\text{r}}_{32}\left({\text{Y}}_{0}-{\text{Y}}_{\text{A}}\right)+{\text{r}}_{33}\left({\text{Z}}_{0}-{\text{Z}}_{\text{A}}\right)\right]}+\;\Delta \text{x′}\\ {\text{y′}}_{\text{a}}={\;y}_{\text{p}}-\text{c}\cdot \frac{\left[{\text{r}}_{21}\left({\text{X}}_{0}-{\text{X}}_{\text{A}}\right)+{\text{r}}_{22}\left({\text{Y}}_{0}-{\text{Y}}_{\text{A}}\right)+{\text{r}}_{23}\left({\text{Z}}_{0}-{\text{Z}}_{\text{A}}\right)\right]}{\left[{\text{r}}_{31}\left({\text{X}}_{0}-{\text{X}}_{\text{A}}\right)+{\text{r}}_{32}\left({\text{Y}}_{0}-{\text{Y}}_{\text{A}}\right)+{\text{r}}_{33}\left({\text{Z}}_{0}-{\text{Z}}_{\text{A}}\right)\right]}+\;\Delta \text{y′} \end{array}$$

where (x′_a_, y′_a_) and (X_A_, Y_A_, Z_A_) represent the coordinates of a generic point A in the image and object space, respectively, (x_p_, y_p_) are the principal point coordinates, (X_0_, Y_0_, Z_0_) are the coordinates of the perspective centre in the object space, r_ij_ (with i,j = 1,2,3) represent the elements of a rotation matrix, c is the principal distance and Δx′ and Δy′ are photo coordinate corrections to the combined (radial and decentring) lens distortion. The combined lens distortion terms can be represented by the equations [12]:

(2) $$\Delta \text{x′}=\Delta x\prime_{rad}+\Delta x\prime_{dec}\;\Delta x\prime =x\prime \frac{\Delta {r\prime }_{rad}}{r\prime }+P_{1}\left({r\prime }^{2}+2{x\prime }^{2}\right)+2P_{2}x\prime y\prime \;\Delta \text{y′}=\Delta y\prime_{rad}+\Delta y\prime_{dec}\;\Delta y\prime =y\prime \frac{\Delta {r\prime }_{rad}}{r\prime }+P_{2}\left(r^{\prime 2}+2y^{\prime 2}\right)+2P_{1}x\prime y\prime \;\Delta r\prime_{rad}=K_{1}r\prime^{3}+K_{2}r^{\prime 5}+K_{3}r\prime^{7}$$

Both camera exterior orientation (defined by X_0_, Y_0_, Z_0_, and r_ij_) and interior orientation (comprising x_p_, y_p_, c, Δx′, and Δy′) are typically obtained through a bundle adjustment [23]. Auspiciously, over the past years, continued advances in digital photogrammetry have increased the number of applications of photogrammetry. In particular, automated image-processing algorithms have attenuated competences needed to deal with photogrammetric projects and therefore, this can certainly be a promising solution for hydraulicians studying certain physical processes with the aid of imaging systems [13]. Having these considerations in mind, the PhotoModeler Scanner software (64 bit) [24] was selected to calibrate the two cameras. PhotoModeler models the radial lens distortions and the decentring distortions through Equations (2). As an output, the software provides some quality indicators (average and maximum residuals) which are extremely useful to judge the overall accuracy of the derived calibration data.

**Figure 6:**
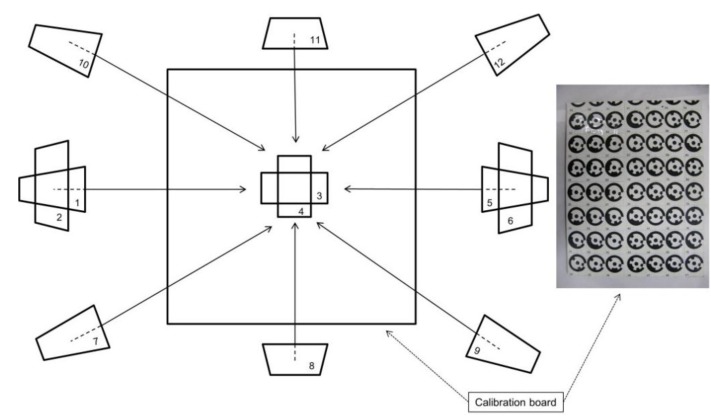
Camera calibration configuration—Note that two distinct environments were considered at this phase: dry and wet (using the plastic water tank) to fully consider the fluid at the camera’s interface during area assessment.

**Figure 7:**
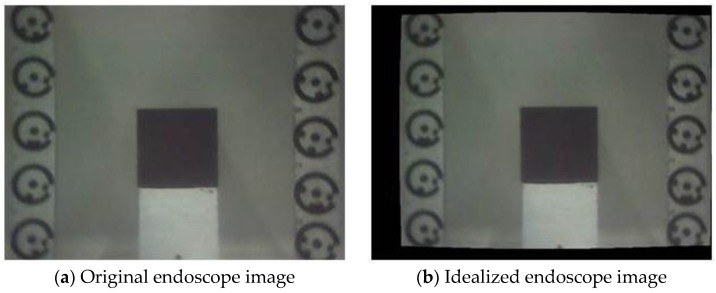
Black metal cube image using the endoscope camera in the plastic water tank.

Figure 6 represents the image configuration (image frames represented by numbers 1 to 12) and the calibration board used to determine the camera calibration parameters for both the D80 camera and the underwater probe (dry and submerged condition for the underwater probe). The calibration board consisted of 49 coded targets generated by PhotoModeler Scanner. Twelve images of the calibration board were captured, with three image frames rotated by 90 degrees (frames 2, 4, and 6 in Figure 6) to provide the possibility to estimate the principal point offset x_p_ and y_p_ of the camera [17,25]. The camera to object distance was set to 0.7 m, the exact same distance used when collecting the metal cube and the bush imagery. The calibration files were subsequently uploaded to a PC, and processed using the camera calibration tool in PhotoModeler Scanner. Finally, camera models determined for the D80 camera and the underwater probe were applied in order to remove the distortion effects of the recorded images. PhotoModeler provides the option to use the estimated camera parameters to produce an undistorted or “idealized” image. In general terms, during idealization, the software re-maps the image pixel by pixel and removes any lens distortion, non-centred principal point and any non-square pixels [24] (Figure 7). The effect of camera calibration on the computation of the surface area will be explored in the following section.

## 4. Results and Discussion

### 4.1. Dry Case

The projected area of a metal cube with known dimensions and a real bush were determined under dry conditions using the endoscope camera and a Nikon D80 DSLR camera normally used for spatial measurement. Table 1 summarizes the estimated cube areas using the two cameras. The first column contains the calibration status of the cameras, whilst the second column tabulates the determined cube area using images acquired with the Nikon camera. The percentage error of the cube area obtained with the Nikon camera is identified in column three and the final two columns represent the cube area and the percentage error determined using the underwater endoscope camera. It should be emphasized that the percentage error was computed as:

(3) $$\left(\frac{\left|\text{areapredicted}-\text{areaactual}\right|}{\text{areaactual}}\right)*100$$

Both cameras achieved similar results when calibrated (percentage error of 0.1%). The Nikon D80 camera attained a percentage error of 0.8% when camera calibration parameters were not considered, whilst the determined percentage error of the underwater endoscope camera was 1.4%. The performance of both cameras is mainly affected by lens distortion, which evidently is of a different magnitude in these two cases. Nevertheless, results demonstrate that both camera lenses are able to derive an accurate area in dry conditions, if appropriately calibrated.

The metal cube was exchanged for the bush and results are presented in Table 2. Obviously, computed areas at this stage can only be compared in relation to each other, as no “true” area estimation is available. Results reinforce the viability of using this particular endoscope camera to obtain accurate estimates of the projected area, once lens distortion is considered. The areas determined varied by 2.7% using images acquired with non-calibrated cameras. Remarkably, areas of similar orders (discrepancy of 0.3%) have been determined using images where lens distortion was accounted for.

**Table 1:** Metal cube area.

Camera Calibration	Area D80 Camera [m^2^]	Error D80 Camera [%]	Area Endoscope Camera [m^2^]	Error Endoscope Camera [%]
Not calibrated dry	0.01063	0.8	0.01040	1.4
Calibrated dry	0.01056	0.1	0.01056	0.1
Not calibrated tank			0.0096	9.0
Calibrated tank			0.0104	1.4
Not calibrated flume			0.0098	7.1
Calibrated flume			0.0107	1.4

**Table 2:** Bush area dry condition.

Camera Calibration	Area D80 Camera [m^2^]	Area Endoscope Camera [m^2^]	Difference D80-Endoscope [%]
Not calibrated dry	0.0324	0.0333	2.7
Calibrated dry	0.0318	0.0317	0.3

### 4.2. Plastic Water Tank

In the presence of static water, distortions are expected to increase since light paths are refracted twice in the vicinity of camera lenses. Again, underwater images of the metal cube were acquired and results are shown in Table 1. Images not corrected for lens distortion exhibit a marked difference to the known metal cube area (error of 9%). However, the percentage error between the computed area and the reference area is reduced to just 1.4% when distortion effects are modelled using the radial lens parameters. This can dramatically reduce the uncertainty of image analysis in these conditions.

The projected area determined for the bush using the endoscope camera image without a lens model diverged from the bush area in dry conditions by 12.3% (Table 3). This error was reduced to just 1.6% when lens distortion was considered. These areas are usually assumed to be coincident since buoyancy effects are taken to be negligible [15]. This is also likely to be true in our case due to the high flexural rigidity of the vegetation stems. Consequently, we hypothesize that this small difference is related to minor experimental errors, e.g., an imperfect alignment of the underwater camera.

**Table 3:** Bush area using the endoscope camera in the plastic water tank.

Camera Calibration	Area Bush Submerged [m^2^]	Area Bush Dry [m^2^]	Difference Submerged-Dry [%]
Not calibrated tank	0.0292	0.0333	12.3
Calibrated tank	0.0312	0.0317	1.6

### 4.3. Open-Channel Flume

This test was conducted under conditions similar to those found in a field environment, especially with respect to water clarity. The water discharge was 0.124 m^3^·s^−1^ and use of the underwater camera had the additional advantage of allowing the adoption of reduced object to camera distances with minimal flow disturbance. For the metal cube, a noteworthy discrepancy was found if calibration is ignored (Table 1). This is visible from the substantial departure from the cube reference area (7.1%). Once again, it is clear that the inclusion of a lens model significantly improves area estimations (from one case to the other, area variation was of 8.4%). A similar conclusion was found for the vegetation specimen (Table 4). If distortion effects are compensated, surface area is actually 5% greater in flow conditions. Moreover, area differences between flowing and dry conditions range from 17.3% (not calibrated) to 5.7% (calibrated). This finding is significant since it expresses morphological adjustment of the specimen due to water flowing over and around the plant in “stressed” conditions.

**Table 4:** Bush area using the endoscope camera in the flume.

Camera Calibration	Area Bush Submerged [m^2^]	Area Bush Dry [m^2^]	Difference Flowing-Dry [%]
Not calibrated flume	0.0284	0.0333	17.3
Calibrated flume	0.0299	0.0317	5.7
Difference calibrated-not calibrated [%]	5.0	4.9	

This trial demonstrated that image acquisition can be problematic in a real river environment. Due to low illumination of the lower parts of the vegetation specimen, external lighting sources had to be used to improve illumination to a suitable level for image processing. Additionally, a high suspended sediment load in the flume appeared to reduce image quality, although not to a level to affect image processing.

## 5. Conclusions

Imaging systems are becoming increasingly used by experimentalists, due to their ability to clarify certain aspects of flow-vegetation interactions. This fact together with the notion that calibration of non-metric cameras is vital to extract reliable spatial data [13,14] inspired the present work. The magnitude of lens distortion depends on the combination of several factors, namely the focus settings of the lens, the camera depth of field, the medium of data acquisition, and the lens itself. In other words, lens distortions and/or refraction effects will always be present, to a greater or lesser extent, when image based approaches are used. By assessing the two most demanding arrangements used in this study (*i.e.*, results obtained with the underwater endoscope in the tank and stressed flow conditions) and considering their worst case scenarios, failure to consider camera calibration would lead to errors of 9.0% and 12.3% (cube and bush in the tank, respectively), 7.1% (cube in the open-channel), and 5% (bush in the open-channel). Distortions are clearly case dependent, whereby a sound calibration procedure such as the one presented here can be highly convenient, since simplistic procedures to evaluate lens distortions magnitude (such as the one suggested by Sagnes [4]) are avoided. Our results illustrate the need to consider these distortion effects explicitly, especially in flume and field studies. This will undoubtedly contribute to the refinement of current experimental practices, particularly on vegetated flows research, which is largely focussed on a laboratory scale. This requirement is expected to be even higher in turbid waters, where short focal distances will be needed to attain optimum results, and consequently larger distortions will be created. Although recognizing the existence of other methods to deal with this subject, e.g., the ray tracing approach [20], the author’s belief that the approach described above constitutes a valuable starting point for experimentalists whenever environmental conditions (e.g., light and turbidity content) are favourable. This can now be accomplished in a relatively straightforward manner by making use of specialized digital photogrammetry tools.
